# Kaposi’s sarcoma herpesvirus latency-associated nuclear antigen broadly regulates viral gene expression and is essential for lytic infection

**DOI:** 10.1371/journal.ppat.1011907

**Published:** 2024-01-17

**Authors:** Shijun Li, Mengbo Wang, Nicholas Van Sciver, Agnieszka Szymula, Vinayak Sadasivam Tumuluri, Athira George, Akshaya Ramachandran, Komal Raina, Catarina N. Costa, Bo Zhao, Majid Kazemian, J. Pedro Simas, Kenneth M. Kaye

**Affiliations:** 1 Departments of Medicine, Brigham and Women’s Hospital and Harvard Medical School, Boston, Massachusetts, United States of America; 2 Program in Virology, Harvard Medical School, Boston, Massachusetts, United States of America; 3 Department of Computer Science, Purdue University, West Lafayette, Indiana; 4 Instituto de Medicina Molecular, Faculdade de Medicina, Universidade de Lisboa, Avenida Professor Egas Moniz, Lisboa, Portugal; 5 Universidade Católica Portuguesa, Católica Medical School, Católica Biomedical Research, Palma de Cima, Portugal; 6 Department of Biochemistry, Purdue University, West Lafayette, Indiana; 7 Broad Institute of Harvard University and Massachusetts Institute of Technology, Cambridge, Massachusetts, United States of America; Hannover Medical School, GERMANY

## Abstract

Kaposi’s sarcoma herpesvirus (KSHV) is a leading cause of malignancy in AIDS and current therapies are limited. Like all herpesviruses, KSHV infection can be latent or lytic. KSHV latency-associated nuclear antigen (LANA) is essential for viral genome persistence during latent infection. LANA also maintains latency by antagonizing expression and function of the KSHV lytic switch protein, RTA. Here, we find LANA null KSHV is not capable of lytic replication, indicating a requirement for LANA. While LANA promoted both lytic and latent gene expression in cells partially permissive for lytic infection, it repressed expression in non-permissive cells. Importantly, forced RTA expression in non-permissive cells led to induction of lytic infection and LANA switched to promote, rather than repress, most lytic viral gene expression. When basal viral gene expression levels were high, LANA promoted expression, but repressed expression at low basal levels unless RTA expression was forcibly induced. LANA’s effects were broad, but virus gene specific, extending to an engineered, recombinant viral GFP under control of host EF1α promoter, but not to host EF1α. Together, these results demonstrate that, in addition to its essential role in genome maintenance, LANA broadly regulates viral gene expression, and is required for high levels of lytic gene expression during lytic infection. Strategies that target LANA are expected to abolish KSHV infection.

## Author summary

Kaposi’s sarcoma herpesvirus (KSHV) is a leading cause of cancer in those with AIDS and current therapies are limited. Like other herpesviruses, KSHV infects cells in two forms, causing latent or lytic infection. KSHV latency-associated nuclear antigen (LANA) is essential for viral latency since it maintains the extrachromosomal viral genome in dividing cells. LANA also maintains latency by antagonizing the KSHV lytic switch protein, RTA, which is capable of triggering lytic replication. Here, we find KSHV lacking LANA is incapable of lytic infection, indicating LANA is required for lytic replication. Further, LANA can have dual effects on viral gene expression. In cells partially permissive for lytic KSHV infection, LANA promoted viral gene expression, but repressed expression in non-permissive cells. Forced RTA expression in non-permissive cells led to LANA switching to promote, rather than repress, most lytic viral gene expression. We found when basal viral gene expression levels were high, LANA promoted expression, while LANA repressed expression at low basal levels. These results show that, in addition to its essential role in genome maintenance, LANA is required for the elevated levels of lytic gene expression that occur during lytic infection. Future approaches that inactivate LANA are expected to abolish KSHV infection.

## Introduction

Kaposi’s Sarcoma (KS)-associated herpesvirus (KSHV or HHV8) is the etiologic agent of KS, the leading AIDS malignancy, primary effusion lymphoma (PEL), and is tightly linked with multicentric Castleman’s disease, a lymphoproliferative disorder [1–4]. Following infection, the linear KSHV genome rapidly circularizes, and persists as an extra-chromosomal episome during latent infection, the default virus state [5]. KSHV encodes ~100 open reading frames (ORFs), of which most are dedicated to lytic virus replication and therefore silenced in latent infection. Only several genes are expressed in latency, including the latency-associated nuclear antigen (LANA/ORF73), a viral cyclin D homolog (v-cyclin/ ORF72), and ORF71/v-FLIP/K13 [6,7].

LANA, an 1162-amino acid protein, mediates persistence of the viral episomal genome, and therefore is required for latency in proliferating cells. Through N-terminal binding to the nucleosome surface of host chromosomes and simultaneous C-terminal binding to KSHV terminal repeat (TR) DNA, LANA tethers KSHV episomes to mitotic chromosomes to ensure segregation of viral genomes to progeny nuclei [8–12]. LANA binding to TR DNA is also necessary to mediate KSHV DNA replication during latent infection. LANA accomplishes this feat through interacting with host cell machinery, including DNA replication factor C (RFC) to enhance proliferating cell nuclear antigen (PCNA) loading [13,14].

In addition to its essential role in viral episome maintenance, LANA exerts a key role in promoting viral latency. The KSHV replication and transcription activator (RTA, ORF50) is the lytic switch protein, and its expression is sufficient to induce expression of lytic KSHV genes, triggering lytic virus infection. LANA represses Rta transcription [15,16] and exerts additional inhibitory effects on RTA to maintain latent viral infection [17]. LANA also interacts with multiple host proteins to exert transcriptional regulatory effects on viral and host genes [18]. For instance, following infection, LANA recruits PRC2 to the viral genome to silence lytic gene expression [19].

Here, we find LANA is required for lytic virus replication. We show LANA exerts context dependent effects, either by promoting or repressing viral gene expression, depending on the basal level of viral gene expression in different cell microenvironments. At low basal expression levels, LANA represses viral gene expression to maintain latency, while at higher levels of basal expression, LANA promotes expression. As a result, in addition to its role in maintaining viral latency, LANA is also necessary for high levels of lytic gene expression during viral replication.

## Results

### KSHV undergoes spontaneous lytic replication in 293 cells

**Fig 1:**
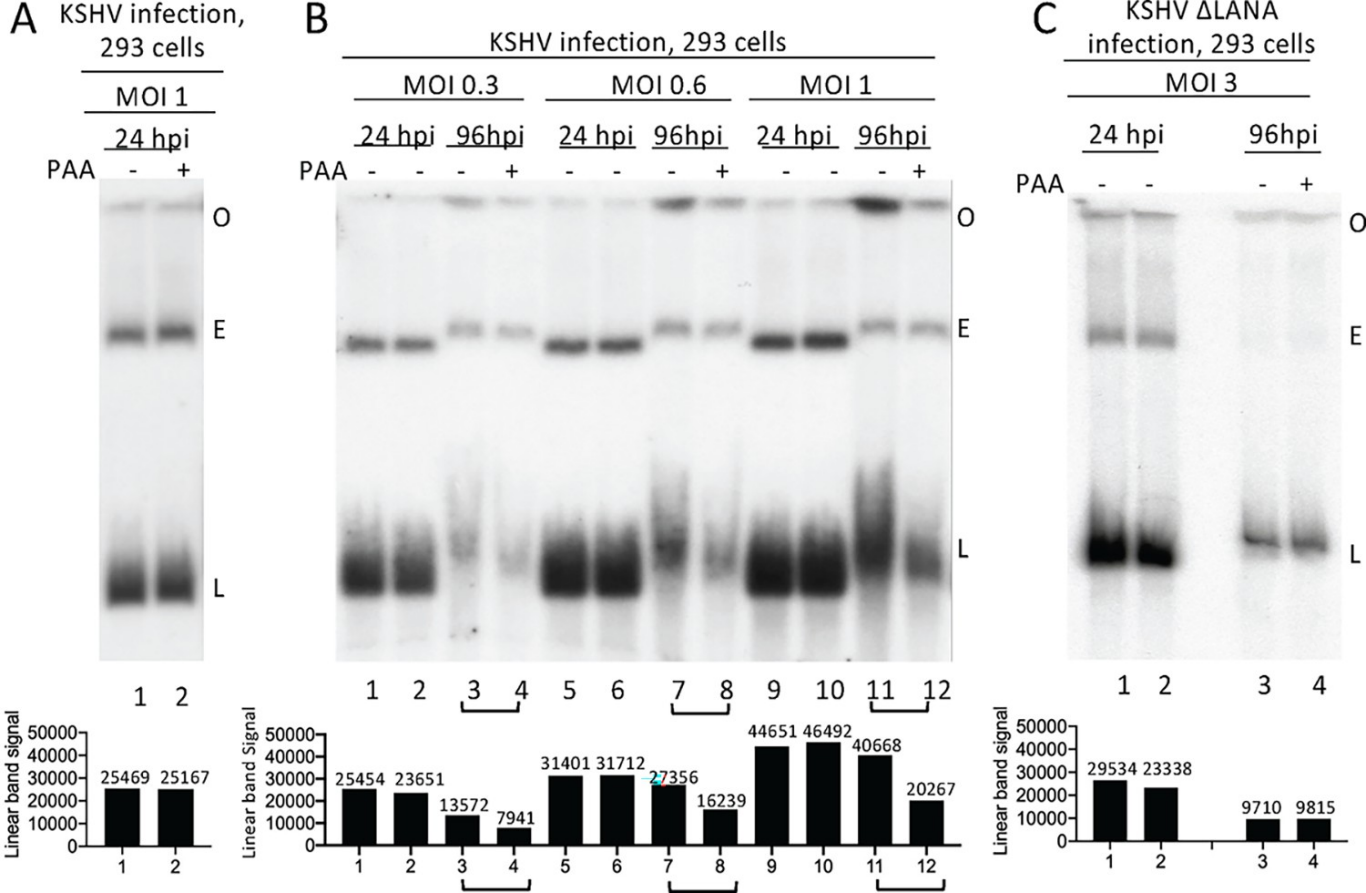
KSHV LANA is required for spontaneous lytic infection in 293 cells. A. Gardella gel of KSHV infected 293 cells at 24 hpi, with or without PAA incubation. B. Gardella gel of KSHV infected 293 cells at the indicated MOI at 24 or 96 hpi with or without PAA. C. Gardella gel following KSHVΔLANA infection at 24 or 96 hpi with or without PAA. Data representative of 2 experiments. A-C. Each lane pair contains independent infections. O, gel origin; E, episomal band; L, linear viral DNA. Linear band signal quantification using Image J is shown below.

We used Gardella gel analysis [20] to assess for lytic KSHV replication following 293 cell infection. In Gardella gels, cells are loaded into wells and are lysed at the start of the gel run. Viral episomal, and linear genomes migrate into the gel and are detected by Southern blot, while chromosomal DNA remains at the gel origin. Episomal genomes are extrachromosomal and circular, and are maintained during latency, while linear viral genomes are generated during lytic virus infection. Linear genomes migrate faster than episomes in Gardella gels, allowing their resolution [21,22].Twenty-four hours following rKSHV.BAC16 (hereafter termed KSHV) [28,68] infection at multiplicity of infection (MOI) 1, both episomal and linear DNA were present (Fig 1A). Incubation with phosphonoacetic acid (PAA), which inhibits viral DNA replication, did not result in decreased linear viral DNA, indicating that the linear genomes at 24 hours post infection (hpi) were from input virus, and not due to lytic replication. At 96 hpi, episomal and linear viral DNA were again present, but, in contrast with 24 hpi, incubation with PAA reduced levels of linear DNA ~2 fold following infection at MOI 0.3, 0.6 or 1.0, indicating lytic DNA replication was occurring (Fig 1B, compare lanes 3 vs. 4, 7 vs. 8, 11 vs. 12). In concert with lytic replication, we observed increased signal at the gel origin (Fig 1B, lanes 3, 7, 11) consistent with viral DNA generated during lytic replication that is too large or complex to enter the gel, such as from concatemers or catenation. Therefore, spontaneous KSHV lytic DNA replication occurs following 293 cell infection.

### LANA is required for lytic replication following 293 cell infection

To assess the role of LANA in lytic replication, we infected 293 cells with KSHVΔLANA [19]. We used MOI 3 rather than a lower MOI because, in the absence of LANA, viral episomes are rapidly lost following cell division. At 24 or 96 hpi, both episomal and linear DNA were present (Fig 1C). In contrast to WT KSHV, PAA did not diminish the level of linear viral DNA, indicating the absence of lytic DNA replication (Fig 1C, compare lanes 3, 4). Therefore, LANA is required for spontaneous KSHV lytic replication following 293 cell infection.

### LANA broadly promotes viral gene transcription in 293 cells

**Fig 2:**
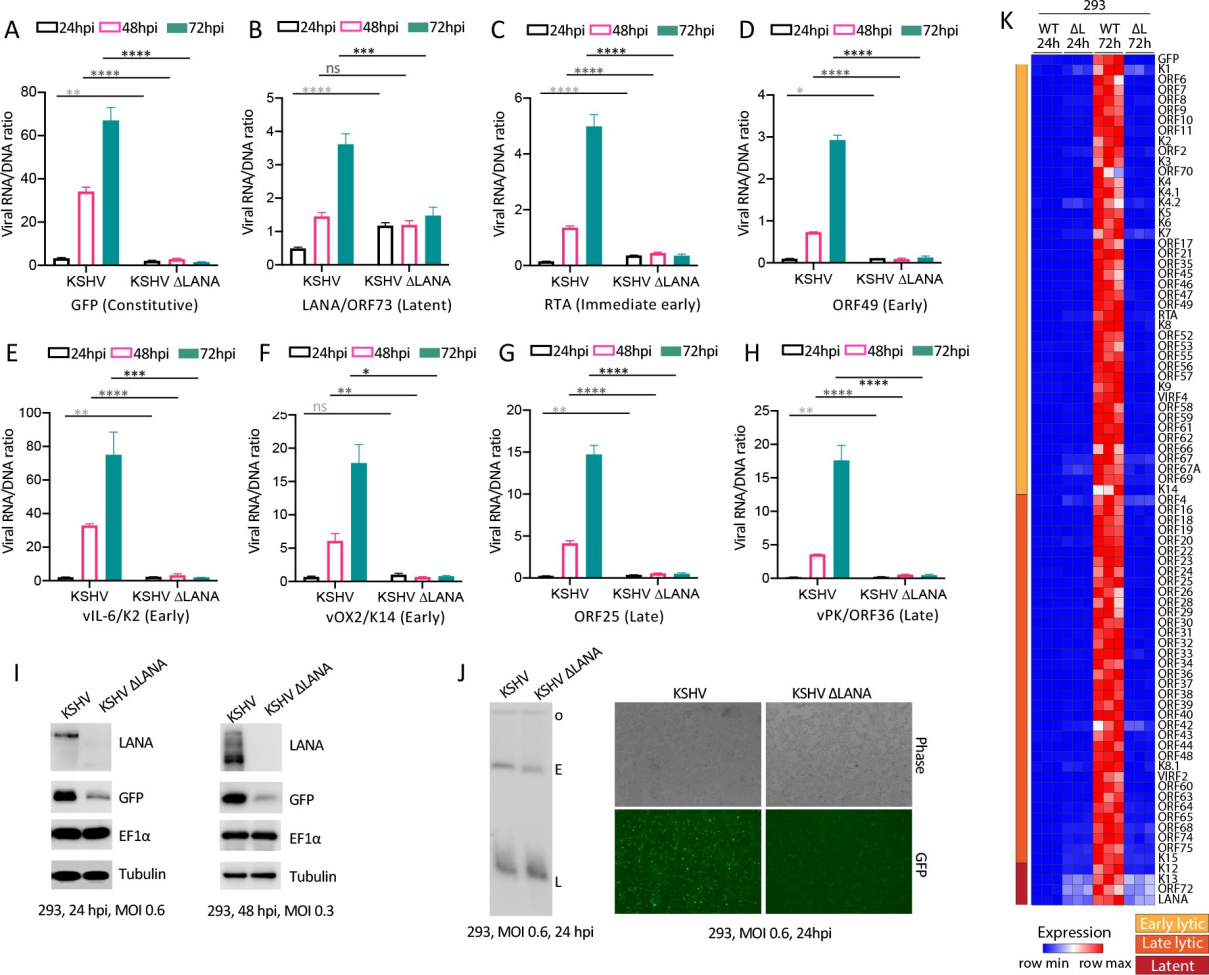
LANA promotes KSHV gene expression in 293 cells. A-H Ratios of viral RNA to DNA following KSHV or KSHVΔLANA infection of 293 cells for the indicated viral genes at 24, 48 or 72 hpi. ns, p>0.05; *, p<0.05; **, p<0.01; ***, p<0.001;****, p<0.0001 by unpaired t test. I. Immunoblot of the indicated gene products following KSHV or KSHVΔLANA infection of 293 cells at 24 or 48 hpi at indicated MOI’s. The multiple LANA bands, which are more prominent at 48 hpi, are due to alternative initiation of translation and an alternative poly A signal.[71,72] GFP is expressed from the EF1α promoter in the recombinant viral genome. J. Gardella gel is shown at left. Phase microscopy (right, top) or detection of GFP expression by fluorescence microscopy (right, bottom) following infection of KSHV or KSHVΔLANA at 24 hpi. Cells were detected using the 4x objective on an EVOS M7000 imaging system (ThermoFisher). K. Heatmap of z score values showing relative viral gene expression for all KSHV genes following KSHV (WT) or KSHVΔLANA (ΔL) at 24 or 72 hpi in 293 cells. Three independent biological replicates are shown for each time point. Early lytic, late lytic, or latent classes of genes are indicated at left.

Since LANA is required for lytic KSHV infection, we asked if LANA regulates viral lytic gene expression. We infected 293 cells with KSHV or KSHVΔLANA at MOI 0.3 and assessed viral gene transcription by real time RT-PCR (Fig 2). To account for variation of levels of viral genomes in cells, we normalized transcription to viral DNA levels (see Methods). At 24 hpi, immediate early gene RTA, early genes ORF49, K2 (vIL6), K14 (vOX2), or late genes ORF25, ORF36, were low following infection with either virus (Fig 2C–2H and S2 Table), consistent with the absence of lytic replication at 24 hpi (Fig 1A). However, at 48 or 72 hpi, expression progressively rose for each of these genes following KSHV infection (Fig 2C–2H and S2 Table), consistent with the lytic replication observed at 96 hpi (Fig 1B). In direct contrast, expression of all lytic genes remained low throughout the experiment following KSHVΔLANA infection (Fig 2C–2H and S2 Table). Therefore, LANA is required for expression of these early and late lytic genes in 293 cells.

We also examined transcription of LANA, a latent gene. KSHVΔLANA has a stop codon inserted after residue 22, allowing the transcript to be assessed despite the absence of full length protein [19]. At 24 hpi, LANA expression was higher in KSHVΔLANA compared to KSHV, suggesting LANA inhibition of its own expression. However, similar to the lytic genes, LANA expression increased at 48 or 96 hpi following KSHV infection (Fig 2B and S2 Table). This finding suggests that higher LANA levels at these later time points leads to transactivating its promoter, consistent with what has been previously described in reporter assays in 293 cells [25–27]. In contrast, and similar to the lytic genes, LANA expression was unchanged throughout the experiment following infection with KSHVΔLANA (Fig 2B and S2 Table).

We also assessed GFP transcription, which is expressed from the host EF1α promoter inserted in the recombinant viral genome [24,28]. Unexpectedly, we found GFP expression rose after KSHV, but not KSHVΔLANA, infection (Fig 2A and S2 Table), similar to the lytic genes and LANA. This LANA effect was specific for the viral genome since the endogenous EF1α level was unchanged following infection of KSHV or KSHVΔLANA (Fig 2I).

To further contextualize our observations, we assessed gene expression for all viral genes at 24 or 72hpi (Fig 2K, S3, S4 and S5 Tables). Similar to results with lytic genes RTA, ORF49, K2 (vIL6), K14 (vOX2), ORF25, ORF36, latent gene LANA, or GFP (Fig 2A–2H), all lytic and latent genes expressed at higher levels following KSHV, as compared to KSHVΔLANA infection, at 72 hpi. Together, these data demonstrate LANA promotes broad viral gene expression following infection in 293 cells, including EF1α promoter driven GFP in the recombinant virus. These findings also suggest absence of KSHVΔLANA lytic virus replication is due to low lytic gene transcription without LANA.

Detection of GFP expression following 293T cell infection is commonly used to determine infectious unit titer of recombinant KSHV stocks. Since GFP expression was decreased in the absence of LANA, it raised the concern that GFP expression following such infection may not reliably reflect the viral titer of KSHVΔLANA. To test this hypothesis, we determined KSHV infectious titer by GFP expression following infection of 293T cells, and then used Gardella gel analyses to normalize KSHVΔLANA viral DNA levels to that of KSHV. Since Gardella analysis demonstrates viral DNA following infection, (and is independent of GFP expression), this approach allows normalization of infectious virus by DNA level. Following such normalization of viral stocks by Gardella DNA levels, we infected 293 cells with MOI 0.6 of KSHV or KSHVΔLANA and detected GFP at 24 hpi. Despite similar levels of infectious virus as assessed by Gardella gel (Fig 2J, left panel), GFP fluorescence was detected at much lower intensity in cells following KSHVΔLANA infection compared to KSHV infection (Fig 2J, right panels). Therefore, to avoid any potential unreliability of using GFP expression to determine KSHVΔLANA infectious units, we used Gardella gel normalization to assess KSHVΔLANA titer throughout this work.

**Fig 3:**
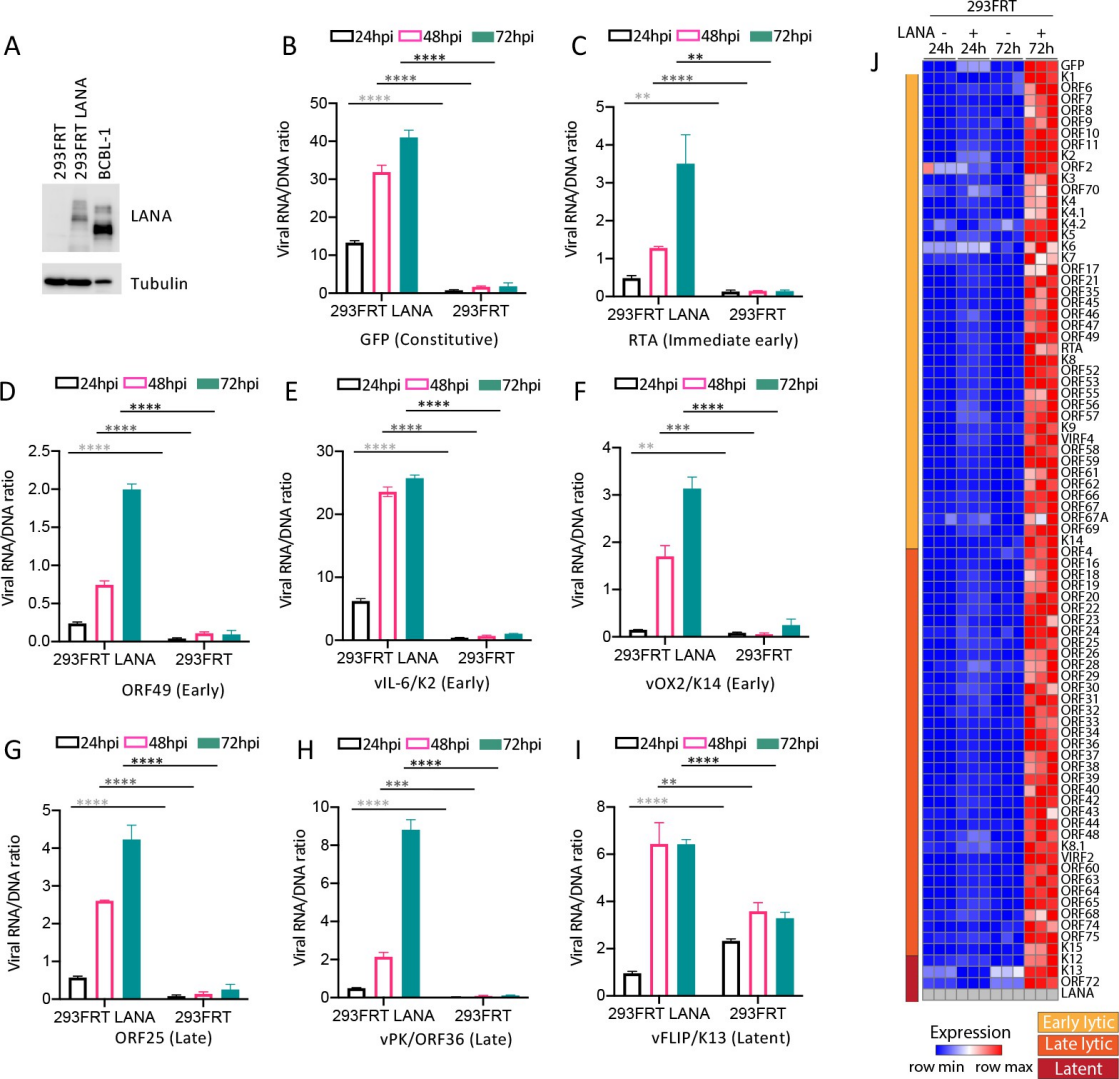
LANA expressed in trans promotes KSHVΔLANA broad viral gene transcription following infection in 293FRT cells. A. Immunoblot of LANA in 293FRT/LANA or BCBL1 PEL cells. 5x10^4^ cells were loaded per lane. (BCBL1 PEL cells have less cytoplasm than do 293FRT cells, accounting for less tubulin in the BCBL1 lane.). B-I. Ratios of viral RNA to DNA following KSHVΔLANA infection of 293FRT/LANA or 293FRT cells for the indicated viral genes at 24, 48 or 72 hpi. **, p<0.01; ***, p<0.001; ****, p<0.0001 by unpaired t test. J. Heatmap of z score values showing relative viral gene expression for all KSHV genes following KSHVΔLANA infection of 293FRT or 293FRT LANA stably expressing cells at 24 or 72 hpi. Three independent biological replicates are shown for each time point. Early lytic, late lytic, or latent classes of genes are indicated at left. Gray color indicates absent values.

We further assessed LANA’s role in viral gene expression with LANA expressed in trans. This approach allowed infection with identical KSHVΔLANA virus of either 293FRT with integrated pcDNA5 vector (hereafter termed 293FRT) or 293FRT cells stably expressing LANA (293FRT/LANA) at levels similar to that of naturally infected BCBL1 PEL cells (Fig 3A). 293FRT cells are 293 cells that contain a single, integrated FRT site that allows targeted insertion of a gene of interest. Similar to results following infection with KSHV (Fig 2), infection of 293FRT/LANA cells at MOI 0.3 resulted in viral gene expression progressively rising for immediate early gene RTA, early genes ORF49, K2 (vIL6), K14 (vOX2), late genes ORF25, ORF36, and also EF1α driven GFP (Fig 3B–3H) at 48 and 72 hpi. Since LANA expression was constitutive from the transgene in the 293FRT/LANA cells, we assessed viral K13 (vFLIP) transcription. vFLIP is a latent gene and is expressed from the same transcript as LANA [7]. Similar to LANA (Fig 2), vFLIP expression rose at 48 hpi, although it did not increase further at 72 hpi (Fig 3I and S2 Table). Following 293FRT infection with KSHVΔLANA at MOI 0.3, expression changed little over time (Fig 3B–3I), similar to results with 293 cells (Fig 2). Analysis of all viral genes at 24 or 72 hpi similarly showed high level gene expression at 72 hpi for all lytic and latent genes only in LANA expressing cells (Fig 3J, S3, S4 and S5 Tables). Therefore, LANA promotes viral gene expression in 293 cells.

### KSHV does not undergo spontaneous lytic replication in NOK or SLK cells

**Fig 4:**
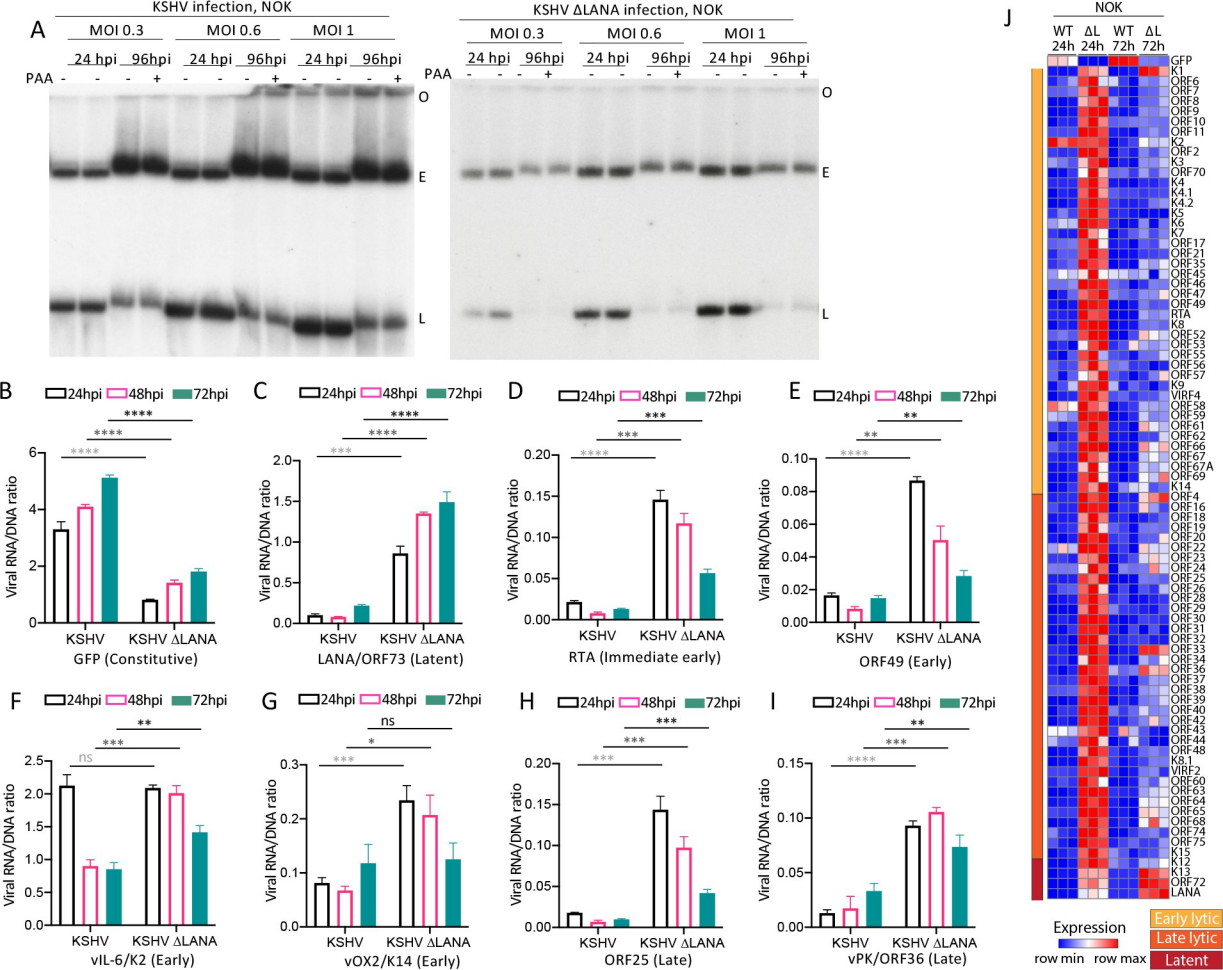
KSHV does not undergo spontaneous lytic infection following infection of NOK cells and LANA inhibits most viral gene expression. A. Gardella gel at 24 or 96 hpi at the indicated MOI following KSHV or KSHVΔLANA infection of NOK cells. Each lane pair contains independent infections. O, gel origin; E, episomal DNA; L, linear viral genomic DNA. B-I. Ratios of viral RNA to DNA following KSHV or KSHVΔLANA infection of NOK cells for the indicated viral genes at 24, 48 or 72 hpi. ns, p>0.05; *, p<0.05, **, p<0.01; ***, p<0.001;****, p<0.0001 by unpaired t test. J. Heatmap of z score values showing relative viral gene expression for all KSHV genes following KSHV (WT) or KSHVΔLANA (ΔL) at 24 or 72 hpi in NOK cells. Three independent biological replicates are shown for each time point. Early lytic, late lytic, or latent classes of genes are indicated at left.

**Fig 5:**
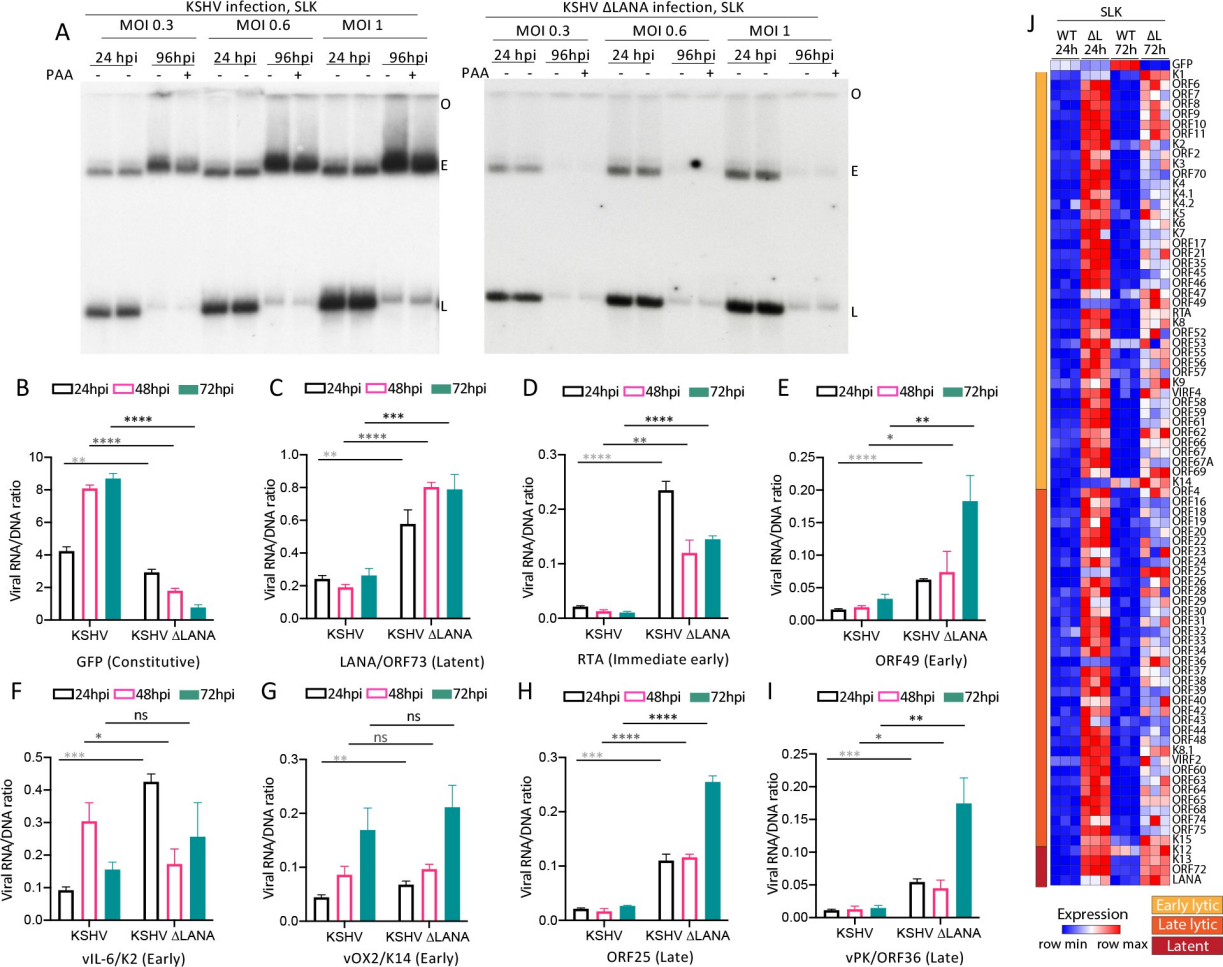
KSHV does not undergo spontaneous lytic infection following infection of SLK cells and LANA inhibits most viral gene expression. A. Gardella gel at 24 or 96 hpi at the indicated MOI following KSHV or KSHVΔLANA infection of SLK cells. Each lane pair contains independent infections. O, gel origin; E, episomal DNA; L, linear viral genomic DNA. B-I. Ratios of viral RNA to DNA following KSHV or KSHVΔLANA infection of SLK cells for the indicated viral genes at 24, 48 or 72 hpi. ns, p>0.05; *, p<0.05, **, p<0.01; ***, p<0.001;****, p<0.0001 by unpaired t test. J. Heatmap of z score values showing relative viral gene expression for all KSHV genes following KSHV (WT) or KSHVΔLANA (ΔL) at 24 or 72 hpi in SLK cells. Three independent biological replicates are shown for each time point. Early lytic, late lytic, or latent classes of genes are indicated at left.

Since KSHV was partially permissive for lytic infection in 293 cells, which exhibit some characteristics of epithelial cells (but also of other cell types) [29,30], and since LANA promoted gene expression in these cells, we wished to further explore KSHV and LANA behavior in additional cell lines that are epithelial. Therefore, we asked if KSHV underwent spontaneous lytic replication following infection of hTERT immortalized normal oral keratinocyte (NOK) [31], or SLK epithelial cells. We infected NOK (Fig 4A) or SLK (Fig 5A) cells with KSHV or KSHVΔLANA at MOI 0.3, 0.6, or 1.0 and performed Gardella gel analyses at 24 or 96 hpi, with or without the addition of PAA at 96 hpi to inhibit lytic replication. In contrast to 293 cell infection (Fig 1B) there was no difference in KSHV linear genome signal with or without PAA incubation at 96 hpi (Figs 4A and 5A), indicating absence of lytic DNA replication. In addition, and as expected, similar to 293 cell infection, there was also no difference in linear genome signal with or without PAA following KSHVΔLANA infection, indicating absence of lytic replication. Therefore, NOK and SLK cells are not permissive for spontaneous lytic replication following KSHV or KSHVΔLANA infection.

### LANA represses viral gene transcription in NOK and SLK cells

We next investigated viral transcription following KSHV or KSHVΔLANA infection of NOK or SLK cells (Figs 4 and 5). Similar to 293 cells, GFP expression progressively increased after KSHV infection (Figs 4B and 5B and S2 Table). However, despite the increasing expression, expression levels were much lower (~10 fold) than in 293 cells. Also similar to 293 cells, GFP expression was lower in the absence of LANA following KSHVΔLANA infection. In contrast to 293 cells, expression of immediate early gene RTA, early gene ORF49, or late genes ORF25, or ORF36, was lower following KSHV compared with KSHVΔLANA infection (Figs 4D–4E, 4H–4I; 5D–5E, 5H–5I and S2 Table), indicating a repressive effect of LANA on these genes. Early gene K2 (vIL6) or K14 (vOX2) expression levels were also lower following KSHV compared to KSHVΔLANA infection at most time points in NOK cells (Fig 4F and 4G), while differences in SLK cells were less evident (Fig 5F and 5G). Notably, K2 (vIL6) or K14 (vOX2) expression level differences between KSHV and KSHVΔLANA were of lower magnitude in both NOK and SLK cells (Figs 4F–4G, 5F–5G and S2 Table) compared to the other lytic genes, suggesting a smaller LANA effect on these genes in these cells. We also examined expression of latency gene, LANA. In contrast to 293 cells and similar to the lytic genes, LANA expression was lower following KSHV compared with KSHVΔLANA infection in both NOK and SLK cells (Figs 4C and 5C and S2 Table), indicating an auto-repressive effect in these cells. Remarkably, other than for GFP, or K2 (vIL6) in NOK cells, expression of genes was very low, with levels typically below one transcript per viral genome, suggesting most genomes are largely silent. Analysis of all viral genes at 24 or 72 hpi similarly showed that, other than for GFP, gene expression was predominantly repressed in KSHV (WT) infected cells (Figs 4J and 5J, S3, S4 and S5 Tables), while KSHVΔLANA (ΔL) gene expression was generally higher. Therefore, in contrast to 293 cells, NOK and SLK cells are not permissive for spontaneous lytic replication following KSHV infection, and LANA repressed, rather than promoted expression of most viral genes.

### LANA is required for lytic replication in SLK cells following induction of RTA expression

**Fig 6:**
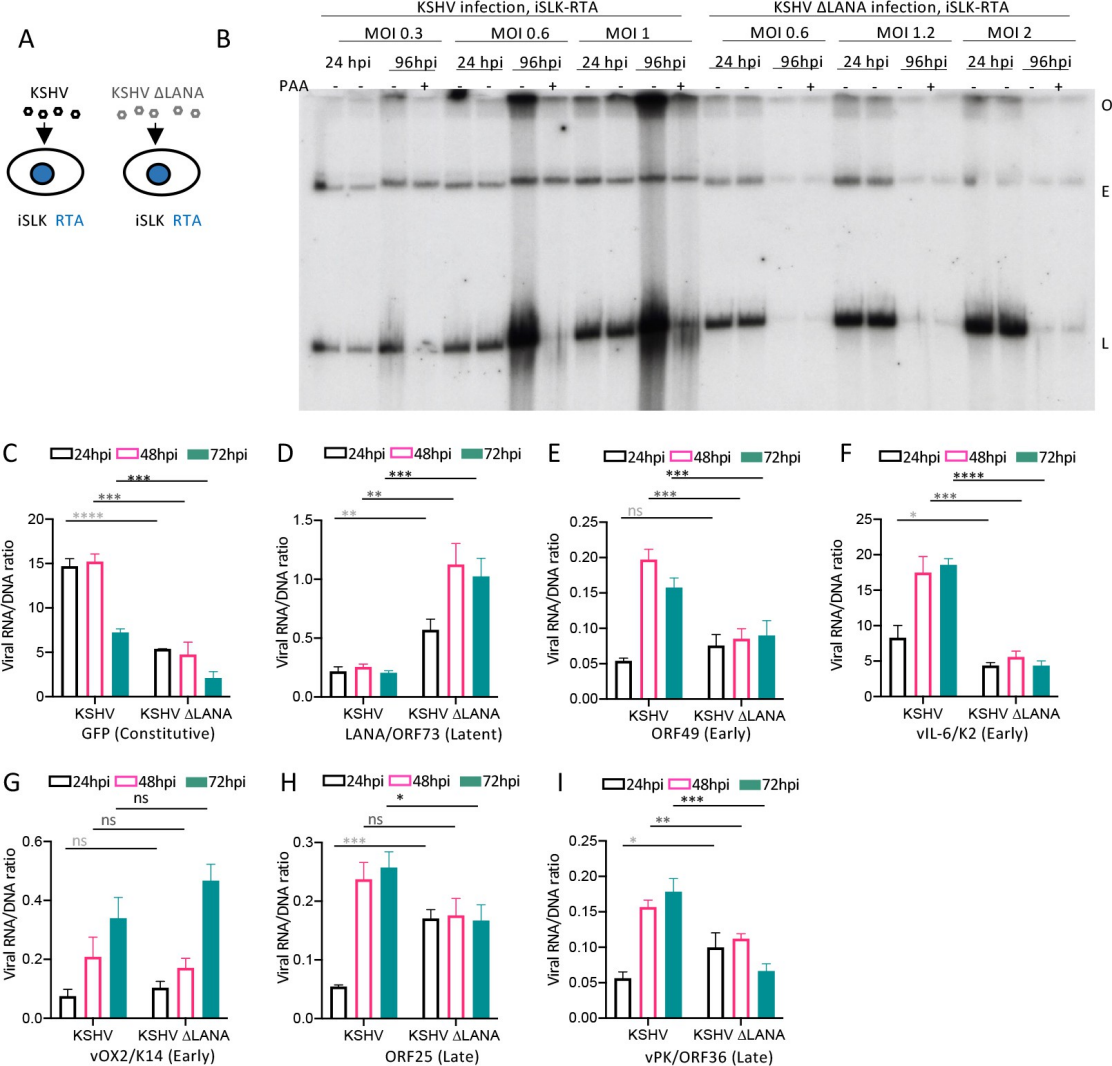
RTA expression in trans induces KSHV, but not KSHVΔLANA, lytic replication in SLK cells and leads to LANA promotion of broad lytic viral gene expression. A. Schematic diagram of KSHV or KSHVΔLANA infection of iSLK RTA cells. B. Gardella gel at 24 or 96 hpi at the indicated MOI following KSHV infection of iSLK RTA cells that were doxycycline induced for RTA expression, with or without PAA. Each lane pair contains independent infections. O, gel origin; E, episomal DNA; L, linear viral genomic DNA. C-I. Ratios of viral RNA to DNA following KSHV or KSHVΔLANA infection of iSLK RTA cells for the indicated viral genes at 24, 48 or 72 hpi. ns, p>0.05; *, p<0.05, **, p<0.01; ***, p<0.001;****, p<0.0001 by unpaired t test.

Since LANA promotes viral transcription in 293 cells following KSHV infection, which spontaneously undergo lytic infection, but represses transcription in NOK and SLK cells, which remain tightly latent, we assessed LANA’s role following induction of RTA expression to induce lytic infection in SLK cells. iSLK cells are SLK cells that contain doxycycline inducible KSHV RTA, the principal KSHV lytic switch protein [23]. Following doxycycline induction of RTA in iSLK cells, we infected cells with KSHV or KSHVΔLANA (Fig 6A). In contrast to KSHV infection of SLK (Fig 5A), Gardella gel analysis demonstrated increased linear viral bands following infection at MOI 0.3, 0.6, or 1.0, which were inhibited at 96 hpi when PAA was included in the incubation (Fig 6B), indicating RTA induction of lytic DNA replication. However, despite RTA induction, no lytic replication occurred following infection with KSHVΔLANA at MOI 0.6, 1.2, or 2.0. Of note, we used higher MOI’s for KSHVΔLANA to better allow detection of viral genomes at 96 hpi, since absence of LANA results in progressive loss of episomal genomes upon cell division (Fig 6B). Therefore, following induction of RTA expression, LANA is required for KSHV lytic replication in iSLK cells.

### LANA promotes viral gene transcription in SLK cells following RTA induction

Since LANA is required for lytic KSHV infection in SLK cells following RTA induction, we asked if LANA promotes viral lytic gene transcription in this setting. We infected iSLK cells induced for RTA with KSHV or KSHVΔLANA at MOI 0.3 and assessed viral gene transcription by real time RT-PCR. In direct contrast to SLK cells, expression of early lytic viral genes ORF49, vIL-6/K2, or late lytic genes ORF25 and vPK/ORF36 was higher at 48 or 72 hpi following KSHV compared to KSHVΔLANA infection (Fig 6E–6F, 6H–6I and S2 Table), indicating LANA promotion, rather than repression, of expression. In fact, LANA promotion of expression of genes from viral genomes following RTA induction may be underestimated here due to the high level of lytic replication (Fig 6B). Underestimation may occur because many of the linear viral genomes may be destined for packaging into virions, and not expressing viral genes, and since expression was determined by RNA/viral DNA ratio, which determines RNA transcript number per viral genome. RTA was not assessed since its transcript was induced from the transgene in the iSLK cells. Other than these lytic genes, and similar to SLK infection, expression of GFP was higher (Fig 6C, S2 Table), LANA lower (Fig 6D, S2 Table), and vOX2/K14 without significant change (Fig 6G, S2 Table) following KSHV compared with KSHVΔLANA infection. These data indicate LANA promotion of GFP expression, auto repression of its own promoter, and minimal effect on vOX2/K14.

**Fig 7:**
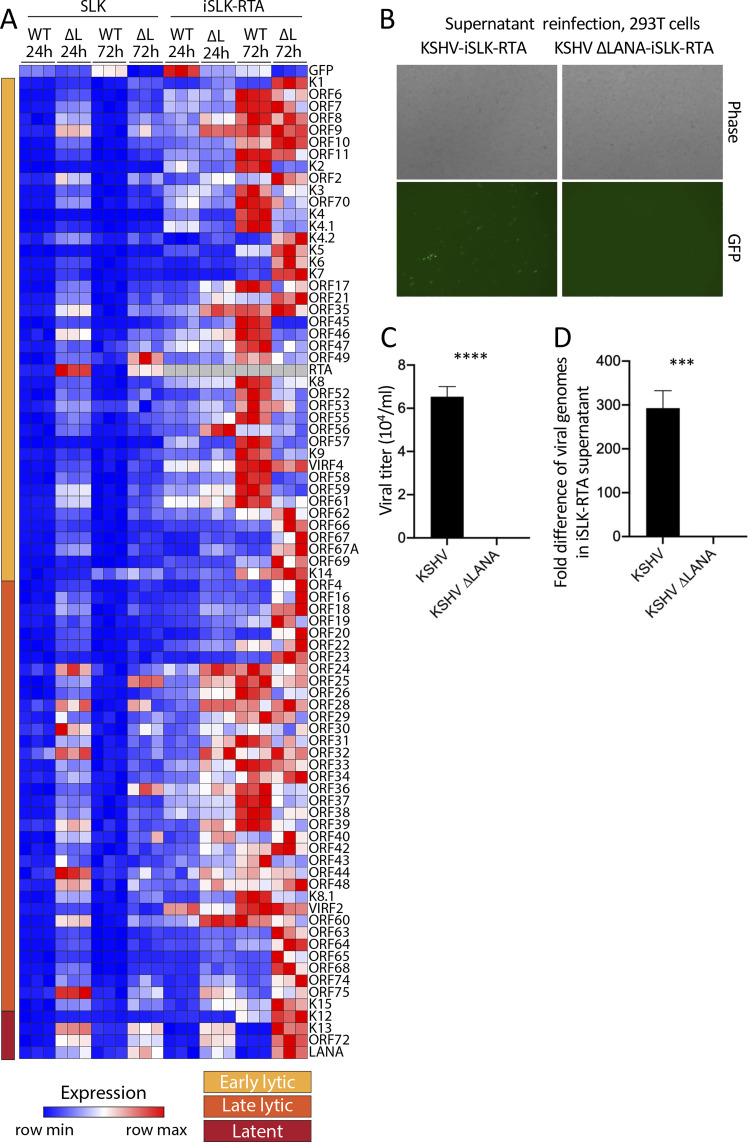
RTA expression in trans leads to LANA promotion of lytic gene expression and virus production in SLK cells. A. Heatmap of z score values showing relative viral gene expression for all KSHV genes following KSHV (WT) or KSHVΔLANA (ΔL) at 24 or 72 hpi in SLK cells (derived from same data as in Fig 5J), or iSLK cells that were induced for Rta expression. Three independent biological replicates are shown for each time point. Early lytic, late lytic, or latent classes of genes are indicated at left. Relative expression values were compared to the mean of all values for both SLK and iSLK for each gene, resulting in different z scores for SLK here compared to Fig 5J, which only included SLK. Gray color indicates absent values. B. Phase (top panels) or fluorescent microscopy (bottom panels) was performed to detect GFP expression from 293T cells 24 hours following incubation with supernatant from KSHV or KSHVΔLANA infected iSLK-RTA cells that had been induced for RTA expression. Cells were detected using the 4x objective on an EVOS M7000 imaging system (ThermoFisher). C. Viral titer of RTA induced, iSLK-RTA cell supernatant, 96 hpi following KSHV or KSHVΔLANA infection. GFP expression was detected by flow cytometry. D. Fold difference in KSHV genome copy number determined by qPCR of RTA induced, iSLK-RTA cell supernatant 96 hpi following KSHV or KSHVΔLANA infection.

We next compared viral gene expression in iSLK RTA induced cells with that of SLK cells across all viral genes (Fig 7A, S3, S4 and S5 Tables). While KSHV infection of SLK cells led to repression of all genes other than GFP at 24 or 72 hpi (same data as in Fig 5J but visually scaled to compare with iSLK), infection of iSLK RTA induced cells led to higher expression for many lytic genes following KSHV compared to KSHVΔLANA infection at 72hpi. Therefore, whereas LANA suppressed most lytic genes following infection of SLK cells, following RTA induction in iSLK cells, LANA actively promoted much lytic gene expression, consistent with its requirement for lytic replication (Fig 6B). This finding shows that forced RTA expression in otherwise nonpermissive SLK cells leads to LANA promotion, rather than inhibition, of expression for many viral lytic genes.

### LANA is required for production of infectious virions following RTA induction in SLK cells

We asked if LANA is required for production of infectious virions following infection of iSLK cells induced for RTA. Following doxycycline induction of RTA in iSLK cells, we infected cells with KSHV or KSHVΔLANA at MOI 1.0. At 96 hpi, 1 mL of cell supernatant was then used to infect 293T cells to assess for presence of virions. GFP, expressed from the recombinant viral genome, was detected in 293T cells after KSHV infection by fluorescence microscopy (Fig 7B), and flow cytometry analysis of GFP expressing cells demonstrated a titer of ~6.5x10^4^ infectious units per mL of supernatant (Fig 7C and S2 Table). In contrast, no GFP positive cells were detected following KSHVΔLANA infection by microscopy or flow cytometry (Fig 7B and 7C and S2 Table), indicating no infectious virions were detected by GFP expression. Since GFP fluorescence detection may underestimate KSHVΔLANA infectious virions due to lower GFP expression in these cells (Fig 2J), we also used real time PCR to detect KSHV or KSHVΔLANA genomes in the supernatant of RTA induced iSLK cells at 96 hpi. Supernatant following KSHV infection had ~300 fold more viral DNA compared to supernatant following KSHVΔLANA infection (Fig 7D). We, therefore, conclude that LANA is required for production of virions following iSLK infection and RTA induction.

## Discussion

In this work, we explored the role of LANA in KSHV lytic infection. Our findings indicate that LANA is required for lytic replication and broadly regulates viral gene expression following primary infection. In both 293 cells (Figs 2 and 3) and iSLK cells induced for RTA expression (Figs 6 and 7), LANA broadly promoted viral expression to drive lytic infection. In contrast, in NOK (Fig 4) or uninduced SLK cells (Fig 5), LANA repressed lytic viral gene expression, and lytic infection was absent. Therefore, LANA can act with dual roles, either enhancing or repressing viral gene expression depending on the cell context. Importantly, when an Rta transgene in iSLK cells was induced to stimulate lytic gene expression in these otherwise non-permissive cells, LANA became an activator, rather than repressor, of viral gene expression, indicating LANA can have dual function within the same cell type (Figs 6 and 7). RTA binds to multiple viral and host sites to induce gene expression and facilitate lytic infection [32], and LANA appears to act in concert with RTA to promote viral gene expression, perhaps by acting at some of the same or complementary target genes.

LANA’s effects varied with basal levels of viral transcription. In 293 cells, the viral transcript to genome ratio was generally at least ~1.0 by 48 hpi, indicating an average of at least one transcript per viral genome. In this setting, LANA promoted viral gene expression. In contrast, in NOK or SLK cells, most viral genes were expressed at viral transcript to genome ratios of less than 0.1, indicating an average of fewer than 1 transcript per 10 viral genomes. In this setting, LANA exerted a repressive effect. However, in contrast to other viral genes, in NOK or SLK cells, GFP, driven from the EF1α promoter in the recombinant virus, expressed at viral transcript to genome levels of greater than 2.0. In this setting, LANA promoted expression, despite simultaneously repressing expression of other viral genes (Figs 4–7). Interestingly, forced RTA expression in iSLK cells (Fig 6) led to LANA promotion of viral gene expression despite some viral transcript to genome ratios remaining less than 1.0, suggesting LANA can act in concert with RTA to promote viral gene expression even at low transcription levels. However, as noted above, LANA promotion of viral gene expression following RTA induction may be underestimated due to the high level of lytic replication (Fig 6B) since many linear viral genomes may be destined for packaging, and not expressing viral genes, and since expression was determined by RNA/viral DNA ratio.

The low transcript to viral DNA genome ratios (often less than 0.1) following KSHV infection of NOK or SLK cells (Figs 4 and 5) suggests most viral genomes are primarily silent following infection. This finding is reminiscent of prior work investigating KSHV epigenetic diversity using single molecule footprinting to characterize chromatin architecture. Results suggested only a fraction of KSHV episomes participate in gene expression or lytic reactivation in PEL cells [33]. Interestingly, LANA expression was also low in NOK and SLK cells, with transcript to viral genome ratios of ~0.1–0.2 (Figs 4C and 5C). Despite these low levels, LANA protein expression is sufficient to maintain viral latency in LANA expressing cells, since LANA is required for KSHV episome persistence [9].

Consistent with our findings, prior studies indicate LANA can promote or inhibit gene expression. In reporter assays, LANA enhanced expression from some promoter constructs such as driven by GAL4 fused with transcription factor Sp1[34], but decreased gene expression from HIV LTR, NF-kB, p53, or pRB dependent gene reporter constructs. [10,27,35–40] GAL4-LANA fusions also repressed the simian virus 40 (SV40) early promoter or HSV thymidine kinase promoter when tethered to adjacent GAL4 binding sites in reporter assays [41]. Further, in LANA expressing BJAB or 293 cells, LANA either activated or repressed expression of ~200, or ~100, respectively, host cell genes [42]. LANA also activated its own promoter in transient reporter assays.[25,27] LANA interacts with a number of host cell partners involved in transcriptional regulation, which may mediate these effects, including CBP [43], mSin3 [44], MeCP2 [45], KDM3A [46], Brd2 [47], MLL family members [48,49], and PRC2 [19]. LANA also inactivates GSK3 to activate β-catenin signaling [50,51], and through GSK3 inhibition, acts to upregulate c-Myc levels [52,53]. Our results suggest the local cell environment, perhaps influenced by availability of the factors mentioned, leads LANA to activate or repress transcription, including from its own promoter. Further, factors such as histone demethylase KDM2B, which rapidly binds to incoming KSHV genomes, leading to RTA down regulation in SLK cells, may be important contributors to the nonpermissive nature of these cells and help establish low basal levels of viral gene expression [54]. It is also possible absence of LANA may lead to altered KSHV chromosome conformation, resulting in changes in viral gene expression. In fact, deficiency in LANA oligomerization induced by mutations at the LANA DNA binding domain dimer-dimer interface led to deficiencies of DNA looping between the KSHV latency and lytic control regions as well as between the TRs and LANA promoter region. These changes were associated with dysregulation of certain KSHV genes [55]. It is possible the absence of LANA could lead to even broader alterations in KSHV chromatin structure that more severely affect viral gene expression.

Results here indicate that LANA can either inhibit (Figs 4D and 5D) or promote RTA expression (Figs 2C and 3C) depending on cell context. Earlier work described a LANA inhibitory role on RTA expression and function. LANA inhibited RTA’s promoter activity in transient reporter assays, and physically interacted with RTA, suggesting two possible mechanisms for RTA inhibition by LANA [17]. LANA interacts with the ORF50/RTA promoter by ChIP [15,56], and LANA repressed ORF50 transcription in latently infected BCBL1 PEL cells, but dissociated from the Rta promoter upon chemical induction of lytic replication [56]. A previous study also showed LANA competes with RTA for regulation of a miRNA that inhibits RTA activity through downregulation of RBPJ, RTA’s critical binding partner [57]. LANA also acts with transcription factor Nrf2 and repressor KAP1 at the Rta promoter to repress Rta transcription [16]. Our findings indicate LANA effects on RTA expression are cell context dependent, indicating nuanced effects in different environments. In fact, LANA promotion of RTA expression may be central to lytic infection, once the decision for lytic infection occurs.

Our finding that LANA is required for KSHV lytic replication differs from prior studies. Genetic disruption of LANA was shown to enhance KSHV lytic gene expression and virus production in 293 cells [56,58], opposite of our findings here. In other work, LANA inhibited the KSHV lytic origin of DNA replication in transient assays [59]. However, LANA also recruits PRC2 to viral lytic gene promoters to repress lytic gene expression, and consistent with our findings, infection of SLK cells with KSHVΔLANA led to higher lytic gene expression, although lytic gene expression was an order of magnitude higher as compared with results here [19].

A difference underlying some of these contrasting findings with our results may relate to the method of normalization used for virus infection. A number of studies used GFP expression from the recombinant viral genomes to normalize infection levels, with the expectation that the host EF1α promoter driving GFP expression would be constitutive [19,56,58]. However, we now find that LANA unexpectedly enhanced GFP expression from recombinant virus (Figs 2–6). As a result, normalization to GFP levels may have led to overestimation of gene expression and viral replication following KSHVΔLANA infection. For instance, GFP expression was ~50 fold higher following KSHV as compared to KSHVΔLANA infection at 72 hpi in 293 cells, and ~10 fold higher in SLK cells. If normalized by GFP, KSHV gene expression and lytic replication would be substantially underestimated, and KSHVΔLANA, overestimated. Instead, we used Gardella gels to normalize infecting levels of viral DNA.

Consistent with our findings, other work using viral DNA levels to normalize KSHV and KSHVΔLANA infection of primary dermal lymphatic microvascular endothelial cells at 2 hpi, also found KSHVΔLANA was deficient for virion production at 96 hpi, suggesting an essential role for LANA in lytic infection [60]. Interestingly, this work also found the latent v-cyclin/ORF72 gene exerted a role in lytic infection [60]. Other work has shown that LANA cytoplasmic isoforms antagonize the innate immune response by acting on the DNA sensor, cGAS, or the cytoplasmic MRN complex, to promote KSHV lytic replication [61,62], and it is possible these effects may contribute to LANA’s ability to promote lytic gene expression and lytic replication observed here. It is also possible that a round of episomal replication, which is LANA dependent, following circularization of incoming viral genomes, is necessary prior to viral genomes being competent for lytic gene expression and replication. However, LANA’s bimodal promotion or repression of lytic gene expression, depending on cell type, suggests LANA’s effects could be at the transcriptional level.

Also consistent with our findings, a related gamma-2 herpesvirus, murine gammaherpesvirus 68 (MHV68), that is null for MHV68 LANA, exhibited less efficient lytic replication following infection of permissive cells at very low MOI. However, LANA from other gamma-2 herpesviruses may function differently, since this work attributed MHV68’s promotion of lytic replication to its destabilization of p53 [63]. In contrast to the MHV68 findings, rhesus rhadinovirus (RRV) LANA inhibited RTA transcriptional activation, and as a result, LANA disruption in RRV resulted in higher levels of lytic viral gene expression and replication in permissive cells [64,65].

Although early genes K2 (vIL6) and K14 (vOX2) were regulated by LANA similar to other lytic genes in 293 cells, LANA had little effect on their expression in NOK or SLK cells (Figs 4 and 5), suggesting they are regulated differently from other genes. In fact, K2 (vIL6) has been described to have latent gene characteristics in some contexts, including SLK cells [66]. The promoter for K14 (vOX2) is located back to back with the latent gene LANA’s promoter, resulting in bidirectional promoters [67], and thus, it is possible this organization may contribute to its differential expression in SLK and NOK cells. Some other lytic genes were also less affected by LANA in NOK cells, especially at 72 hpi (e.g. ORFs 28–32) (Fig 4J and S3 Table), suggesting potential differential regulation in these cells.

We observed increasing MOI resulted in increased levels of lytic replication in 293 cells (Fig 1), which were spontaneously permissive for lytic infection. Similar results were reported following infection of primary human dermal lymphatic microvascular endothelial cells [60]. It is possible increased numbers of virions entering cells at higher MOI leads to higher LANA expression and greater promotion of lytic gene expression in the subset of cells permissive for lytic infection.

In summary, LANA is required for lytic replication and exhibits dual, context dependent, effects, either promoting or repressing expression of viral genes. These characteristics suggest a high level of complexity through which LANA interacts with host cell machinery to broadly regulate viral gene expression in a cell type dependent fashion.

## Materials and methods

### Cell lines

SLK (NIH AIDS Reagent Program), HEK293 or 293T (293 or 293T, ATCC) and Flp-In 293 T-REx (293FRT, Invitrogen) cells were maintained at 37°C in Dulbecco’s Modified Eagle’s Medium (DMEM; Gibco-BRL) supplemented with 10% (vol/vol) bovine growth serum (BGS)(Hyclone) and 15 μg/ml gentamicin. iSLK BAC16 cells [68] (kind gift of Rolf Renne) contain integrated, doxycycline-inducible, KSHV replication and transcription activator (RTA), and were maintained at 37°C in DMEM supplemented with 10% (vol/vol) BGS and 15 μg/ml gentamicin. 1mg/ml hygromycin in combination with 1mg/ml G418 was used to maintain KSHV BAC16, and the rtTA Tet-On transactivator (RTA), respectively. The iSLK LANA KO BAC16 cell line [19] carrying constitutively expressed LANA, LANA null KSHV BAC16 and doxycycline-inducible RTA was a gift from Zsolt Toth (University of Florida). The iSLK LANA KO BAC16 cells were maintained at 37°C in DMEM supplemented with 10% (vol/vol) BGS and 15μg/ml gentamicin, 10 μg/ml blasticidin, 1mg/ml hygromycin and 1mg/ml G418. Blasticidin, hygromycin and G418 were used to maintain LANA, LANA KO KSHV BAC16, and RTA respectively.

hTERT-immortalized oral epithelial NOK cells [31] (kind gift of Karl Munger) was grown in Keratinocyte SFM supplemented with 0.1 μg epidermal growth factor (EGF) and 12.5 mg bovine pituitary extract (BPE) per 500 ml media (Gibco 17005042).

iSLK-RTA cells [23] were kindly provided by Dirk Dittmer.

### Plasmids and cloning

pcDNA5/FRT/TO (pcDNA5) and pOG44 plasmids were obtained (Invitrogen.) pOG44 is the Flp-recombinase expression vector designed for the Flp-In System to integrate the pcDNA5/FRT/TO vector containing the gene of interest under control of a tetracycline regulated promoter into the genome of 293FRT cells via Flp Recombination Target (FRT) sites. pcDNA5- ZZ-LANA-FLAG plasmid [14] (which contains HindIII-ZZ-BamHI-EcorV-LANA-NotI-3xFLAG) was digested with HindIII and EcoRV to remove the ZZ tag. The polylinker sequence between HindIII and EcoRV from pcDNA5/FRT/TO was then inserted into these HindIII and EcoRV sites to generate pCDNA5 LANA-3XFLAG. pEGFP-C1-LANA 779–1162 contains the indicated LANA residues downstream of GFP in the pEGFP-C1 vector (Clontech). GFP LANA 933–1162 was previously described [69].

### Generation of stable cell lines

Stable 293FRT cell lines were obtained according to the manufacturer’s protocol (Invitrogen). Briefly, 1 μg of pOG44 (Invitrogen) was co-transfected with 0.5 μg of pcDNA5 or pcDNA5 LANA-Flag into 2×10^6^ 293FRT cells using lipofectamine 2000 following the manufacturer’s instruction (Thermo Fisher Scientific). The transfected cells were incubated with 50 μg/mL hygromycin to select for pcDNA5 or pcDNA5 LANA-FLAG (both of which contain the FRT recombination site for targeted integration). After 3 weeks of hygromycin selection, single colonies were picked and screened using western blot. The pcDNA5 control or pcDNA5 LANA-FLAG expressing 293FRT cell lines were expanded and used for experiments. Since the BGS in the culture medium contains low levels of tetracycline, further doxycycline induction for LANA expression was not necessary, and the basal LANA expression in 293FRT cells was at a level similar to that of BCBL1 PEL cells as determined by immunoblot.

### KSHV production

WT KSHV and LANA null KSHV were prepared as previously reported. rKSHV.BAC16 was generated by replacing the RFP-GFP-Puro resistance cassette of rKSHV.219 [24] with a loxP-flanked GFP-Hygro resistance cassette containing BAC vector element [28]. A stop codon was inserted into rKSHV.BAC16 after amino acid 22 of ORF73 to generate LANA null rKSHV.BAC16 [19]. No downstream initiated LANA products were observed following 293 cell infection with this virus (S1 Fig). Both WT and LANA null KSHV constitutively express hygromycin B phosphotransferase gene and GFP. WT KSHV was generated from the iSLK cells bearing rKSHV.BAC16 and inducible RTA. LANA null KSHV was generated from the iSLK cells bearing LANA null rKSHV.BAC16 with inducible RTA. The cells maintain the LANA null genome due to LANA expression from a stably integrated LANA expression cassette. RTA expression in iSLK.BAC16 or iSLK LANA null BAC16 cells was induced with 1 μg/ml doxycycline, and cells were also incubated with 20 ng/ml tetradecanoyl phorbol acetate (TPA) and 1mM sodium butyrate (NaB) for 24 h to activate the viral lytic cycle. Cells were then maintained in fresh media without doxycycline, TPA or NaB for an additional 4 days. KSHV in the supernatant was filtered through a 0.45 μm membrane, collected by ultracentrifugation at 25,000g for 4 h, and then resuspended in 20 mL fresh DMEM medium containing 10% BGS.

### Viral infections

0.4×10^6^ of 293 or 293FRT cells/well were plated in 12 well plates. 0.2×10^6^ of SLK or iSLK-RTA, or 0.5×10^6^ of NOK cells/well were plated in 6 well plates. iSLK-RTA cells were supplemented with 1 μg/ml doxycycline to induce RTA expression at the time of cell plating. Cells were infected when they reached ~80% confluence, generally the day following plating. Cells were infected at the indicated MOI by 1 h of spinoculation at 2000g at room temperature. At 24 hpi, the culture medium containing virus was exchanged for fresh culture medium (without virus).

For Gardella analyses, infected cells from two independent wells were each collected at 24 hpi. Cells from one well were harvested for Gardella gel, and cells in the remaining well were seeded into a well of a 6 well plate for growth in the presence or absence of sodium phosphonoacetate (PAA) (Sigma Aldrich) to inhibit viral lytic DNA synthesis. Cells were then collected at 96 hpi for Gardella gel analyses. PAA was added to the medium at 300 μM either at the time of infection when assessing for lytic replication within the first 24 hpi, or at 24hpi when assessing for lytic replication between 24 and 96 hpi.

For qPCR analyses, infected cells were collected at 24 hpi and half were used for qPCR. Of the remaining half, two-thirds were seeded per well in wells of a 6 well plate and then harvested at 48 hpi. The other third was seeded per well in wells of a 6 well plate and then harvested at 72 hpi for qPCR analysis.

### Gardella gel analysis

Infected cells were assessed for latent and lytic replication by Gardella gel [20]. Cells were loaded in agarose gel wells containing sodium dodecyl sulfate and pronase protease (53702, Millipore). Cell lysis occurs as electrophoresis begins in the Tris borate-EDTA buffer. The gel was run overnight at 110V, and DNA was transferred to a nylon membrane. Episomal and linear viral DNA was detected by a ^32^P-labeled KSHV TR probe. Image J was used to quantify the intensity of the linear genomic bands on Gardella gels.

### Fluorescent microscopy

At 24 hpi, GFP expressing cells were detected using the 4x objective on an EVOS M7000 imaging system (ThermoFisher).

### SDS-PAGE, western blotting and transfections

Proteins were resolved by SDS-PAGE and transferred to nitrocellulose membranes for western blot. Membranes were incubated with the primary antibody in PBS containing 0.05% Tween 20 (PBST) and 0.5% (weight/volume) non-fat dry milk at 4°C overnight followed by incubation with secondary antibody in PBST at room temperature for 1 hour. The following antibodies were used at the indicated dilutions: anti-GFP (632380, Clontech, 1/5000), anti-LANA immune serum (1/5000), anti-tubulin (66031-1-lg, Proteintech, 1/5000) anti-EF1α (05–235, Millipore, 1/5000). Secondary antibodies used were goat anti-mouse IgG HRP (1030–05, Southern Biotech, 1/5000) and goat anti-human IgG-HRP (2010–05, Southern Biotech, 1/5000).

For transfections, ~0.5 μg of GFP LANA 779–1162 or GFP LANA 933–1162 plasmid was used to transfect ~0.5x10^6^ 293 cells in one well of a 12 well plate, at which point cells were at ~70–80% confluence, using Lipofectamine 3000 (L3000015, Invitrogen) according to the manufacturer’s instructions. Transfected cells were harvested at 48h post transfection.

### Quantitative RT-PCR determination of expression levels

To assess viral gene expression, ~1×10^6^ cells were harvested at the indicated time points. Half the cells were used for RNA extraction and the other half for DNA extraction by the method of Hirt [70]. Total RNA was isolated from 0.5×10^6^ cells using Trizol as per the manufacturer’s instructions (Thermo Fisher Scientific) and incubated with RQ1 RNAse-free DNAse (Promega) to digest any residual DNA. RNA was dissolved in 100 or 200 μl (V^total RNA^) of nuclease-free water (Thermo Fisher Scientific) and ~10 μl (V^RT RNA^) RNA was reverse transcribed in 20 μl reaction volumes using iScript Reverse Transcription Supermix (1708841, Bio-Rad) to generate cDNA. The resulting cDNA was diluted to 100 μl in nuclease-free water. Viral DNA from 0.5x10^5^ cells was prepared by Hirt DNA extraction [70]. The DNA was then dissolved in 200 μl of nuclease-free water. Quantification was performed by real-time quantitative PCR (qPCR) using Power SYBR Green PCR Master Mix (Thermo Fisher Scientific). The final reaction volume was 20 μl, containing 2 μl of the template and 100 nM of each primer specified. Primer sequences used are listed in S1 Table. The same primer pairs were used for each cDNA or DNA amplification comparison for GFP, LANA, K13, RTA, ORF49, K2, K14, ORF25, or ORF36. Real time amplification was performed using a QuantStudio 3 (Thermo Fisher Scientific) according to the manufacturer’s instructions. All samples were run in triplicate. Mock samples without the addition of reverse transcriptase were included as negative controls using GFP primers for all qPCR and also for qualitative PCR (S2 Fig). The relative viral transcript level to viral DNA level was calculated by (2^-(CtcDNA-CtDNA)^* (cDNA dilution factor)*V^total RNA^/ V^RT RNA^)/(volume total DNA/volume DNA in PCR reaction).

To determine the relative viral transcript to viral DNA level of KSHV genes other than GFP, LANA, K13, RTA, ORF49, K2, K14, ORF25, or ORF36, cDNA expression values were normalized against each of 7 different viral DNA amplifications (GFP, LANA (or K13 for Fig 3J), ORF36, ORF25, ORF49, K2, or K14) using the ΔCT method above to obtain relative expression values. Subsequently, for each experiment (293, 293FRT, NOK, SLK), and each KSHV gene, the relative expression values of KSHV and KSHVΔLANA experimental time points were transformed into z-scores. The z-score is calculated by taking the difference between the relative expression value and the mean and then dividing it by the standard deviation. The mean and the standard deviation of the relative expression values were calculated for each gene across the samples for that experiment. For the combined SLK/iSLK heatmap, the mean and the standard deviation were determined across all SLK and iSLK samples. This approach yields 7 z scores for each gene in each sample relating to the 7 DNA probes (above) used for normalization. The final expression value was then calculated as the average of these 7 z-score values and used for heat map generation.

### Viral titration

KSHV or KSHV ΔLANA were titrated differently. For KSHV, serial volumes ranging from 50 μl to 300 μl were used to infect 1×10^6^ of 293T cells in 12 well plates. At 24 hpi, the percentage of GFP positive cells (expressed from the recombinant KSHV) were assessed by flow cytometry using a CellQuest Pro (BD FACSCalibur). MOI ~1 was determined as the concentration of virus leading to ~60% of KSHV infected 293T cells expressing detectable GFP.

Since GFP expression is lower following KSHVΔLANA infection compared to after KSHV infection (Fig 2A, 2I and 2J), we normalized KSHVΔLANA virion DNA content to that of KSHV using Gardella gel analysis at 24 hpi to achieve similar levels of episomal and linear DNA. (Twenty-four hpi was used to avoid loss of KSHVΔLANA episomes at later time points due to cell division in the absence of the LANA episome maintenance protein.) For KSHVΔLANA titration, serial volumes of KSHVΔLANA ranging from 50 μl to 300 μl or KSHV at MOI 1 were used to infect 1×10^6^ of 293T cells in parallel 12 well plates. At 24hpi, cells from each infection were collected and analyzed by Gardella gel. The KSHVΔLANA virus amount used for infection that resulted in the same virus genome signal on Gardella gel as did KSHV infection at MOI 1, was considered to be MOI 1 for KSHVΔLANA.

To assess viral titer following KSHV or KSHVΔLANA infection of iSLK-RTA cells, iSLK-RTA cells were infected as described above. Twenty-four hours following infection, medium was removed, and cells washed three times with PBS (Gibco, 14190–144) by gently pipetting off the supernatant, and then fed with 3 mL fresh complete DMEM medium containing 1 μg/ml doxycycline. Supernatants of the KSHV or KSHVΔLANA infected iSLK-RTA were collected at 96 hpi. Virus in supernatants was assessed by infecting 293T cells or qPCR. 1x10^6^ 293T cells were infected with 1 mL of supernatant by 1 h of spinoculation in 12 well plates. At 24 hpi, GFP positive cells were imaged with the 4x objective on an EVOS M7000 imaging system (ThermoFisher), and, where indicated, percentages of GFP positive cells were analyzed with flow cytometry using CellQuest Pro (BD FACSCalibur). The infectious unit viral titer per mL of supernatant was then calculated per 1 mL using the flow cytometry data. Viral genomes in the supernatant were also quantified by qPCR in a 20 μl reaction volume after adding 2 μl of neat supernatant template directly to the mix, which included 100 nM GFP primers. Three independent experiments were performed. The Ct value of KSHV or KSHV ΔLANA infected iSLK-RTA supernatant was recorded as Ct KSHV or Ct KSHVΔLANA respectively. The relative fold difference of viral genomes compared to that of KSHVΔLANA in iSLK-RTA supernatants was calculated by 2^-(Ct KSHV or Ct KSHVΔLANA-Ct KSHVΔLANA(mean))^.

### Statistical analysis

Statistical analyses were performed using GraphPad Prism 7 (GraphPad Software Inc.). Specific details for analyses are described in the figure legends. Two tailed, unpaired t tests were used to compare two populations. In all cases: ns indicates P > 0.05, * indicates P<0.05, ** indicates P<0.01, *** indicates P<0.001, **** indicates P<0.0001.

## Supporting information

S1 Fig KSHVΔLANA does not express truncated LANA products from downstream initiation of translation. Immunoblot was performed using anti-LANA or anti-GFP antibody following infection of 293 cells with KSHV or KSHVΔLANA, or following transient tranfection of GFP LANA 779–1162 or GFP LANA 933–1162 in 293 cells. LANA, GFP, GFP LANA 779–1162 or GFP LANA 933–1162 bands are indicated by asterisks to the right of each band. GFP is expressed from the recombinant KSHV or KSHVΔLANA genomes. Control lane contains uninfected and untransfected 293 cells. GFP LANA 779–1162 contains LANA repeat elements, accounting for increased signal with the LANA polyclonal antibody compared to GFP LANA 933–1162, despite its lower expression, as observed in the anti-GFP immunoblot.
(TIF)

S2 Fig PCR amplification of RNA following KSHV or KSHVΔLANA infection in the absence of reverse transcriptase. To assess for residual DNA in RNA preparations, samples were PCR amplified using GFP primers. Positive control DNA template lanes are indicated, and contain PCR reaction products following addition of DNA extracted from iSLK.BAC16 cells (which are infected with BAC16 KSHV that contains recombinant GFP.) Amplified product is 200bp. 100 bp DNA ladder size markers (Invitrogen) are indicated. Gels were cut at marker lanes to allow imaging of the entire gel as gels were otherwise too large to be accommodated in the imager.
(TIF)

S1 Table Oligonucleotides used for PCR amplification. (XLSX)

S2 Table Data supporting Figures 2–7. (XLSX)

S3 Table Data supporting heatmaps in Figures 2, 3, 4, 5 and 7. (XLSX)

S4 Table qPCR Ct values following amplification of viral cDNA used in heatmap generation. (XLSX)

S5 Table z scores used for heatmap generation. (XLSX)
