# Supplementary figures and images for: Kaposi’s sarcoma herpesvirus latency-associated nuclear antigen broadly regulates viral gene expression and is essential for lytic infection
Source: PLoS Pathog. 2024 Jan 17;20(1):e1011907. doi: 10.1371/journal.ppat.1011907 (PMC10793894; doi:10.1371/journal.ppat.1011907)

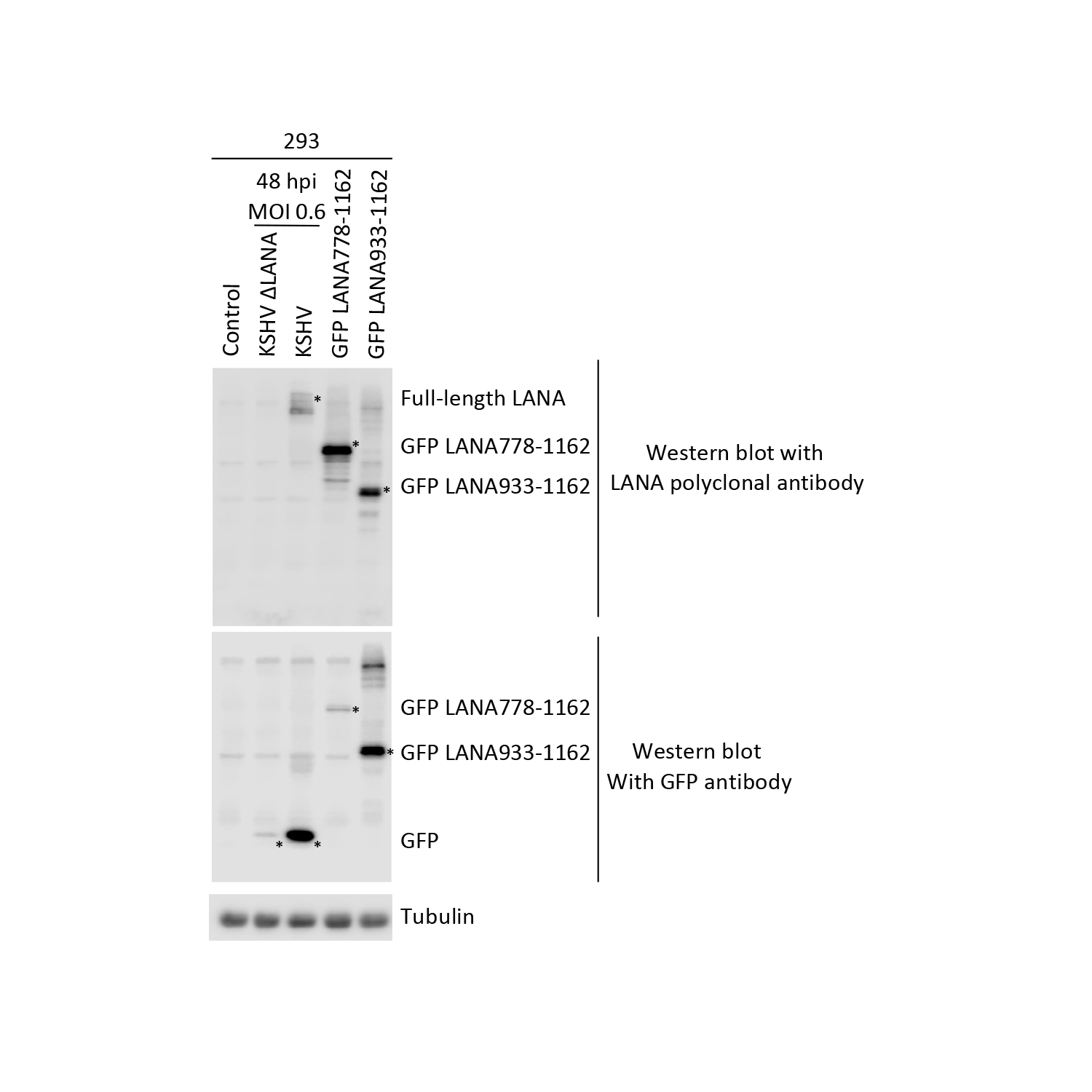

Supplement: S1 Fig — Immunoblot was performed using anti-LANA or anti-GFP antibody following infection of 293 cells with KSHV or KSHVΔLANA, or following transient tranfection of GFP LANA 779–1162 or GFP LANA 933–1162 in 293 cells. LANA, GFP, GFP LANA 779–1162 or GFP LANA 933–1162 bands are indicated by asterisks to the right of each band. GFP is expressed from the recombinant KSHV or KSHVΔLANA genomes. Control lane contains uninfected and untransfected 293 cells. GFP LANA 779–1162 contains LANA repeat elements, accounting for increased signal with the LANA polyclonal antibody compared to GFP LANA 933–1162, despite its lower expression, as observed in the anti-GFP immunoblot. (TIF) [file ppat.1011907.s001.tif]

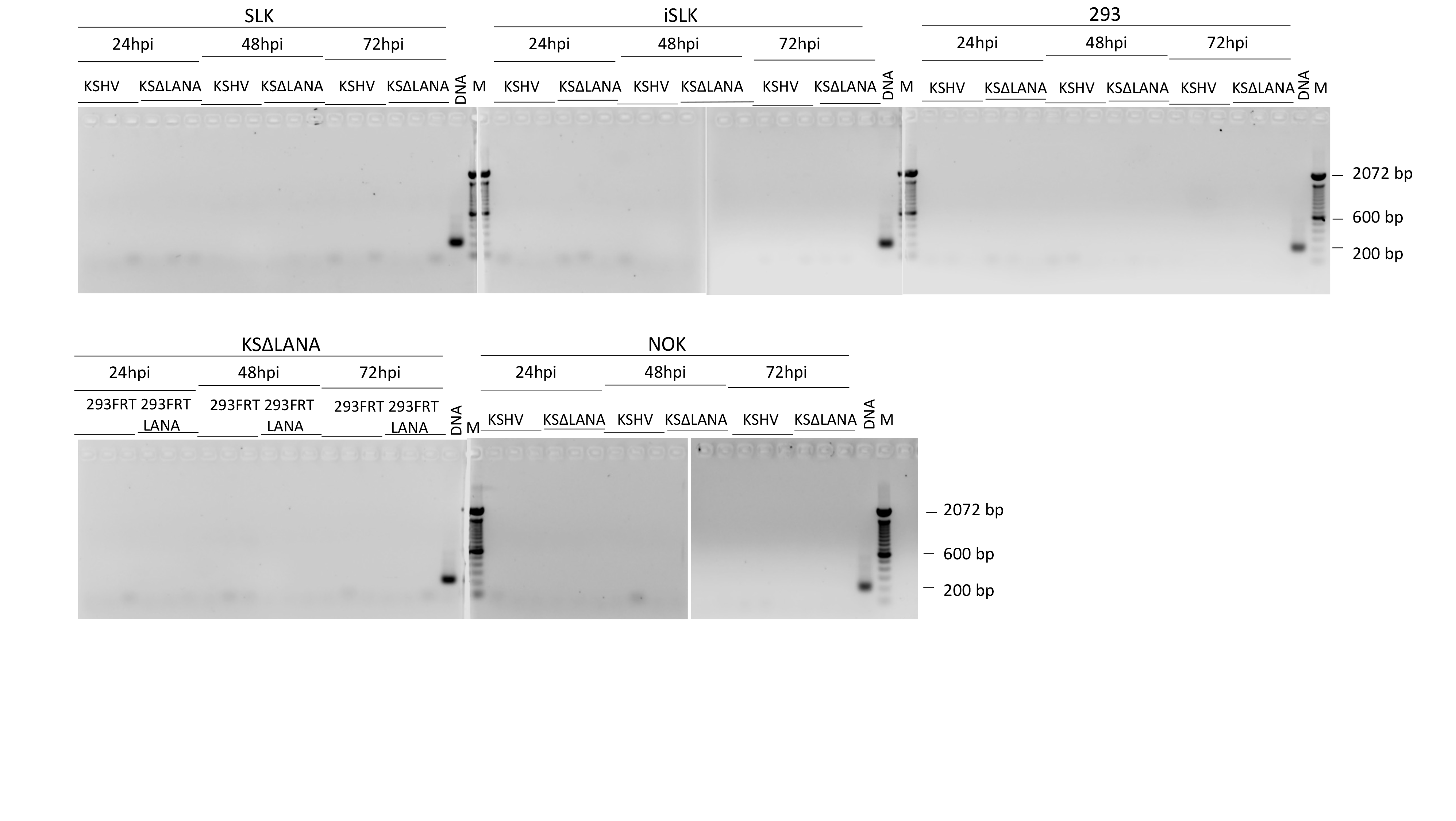

Supplement: S2 Fig — To assess for residual DNA in RNA preparations, samples were PCR amplified using GFP primers. Positive control DNA template lanes are indicated, and contain PCR reaction products following addition of DNA extracted from iSLK.BAC16 cells (which are infected with BAC16 KSHV that contains recombinant GFP.) Amplified product is 200bp. 100 bp DNA ladder size markers (Invitrogen) are indicated. Gels were cut at marker lanes to allow imaging of the entire gel as gels were otherwise too large to be accommodated in the imager. (TIF) [file ppat.1011907.s002.tif]
